# Machine learning classification of archaea and bacteria identifies novel predictive genomic features

**DOI:** 10.1186/s12864-024-10832-y

**Published:** 2024-10-14

**Authors:** Tania Bobbo, Filippo Biscarini, Sachithra K. Yaddehige, Leonardo Alberghini, Davide Rigoni, Nicoletta Bianchi, Cristian Taccioli

**Affiliations:** 1 Institute for Biomedical Technologies, National Research Council (CNR), Via Fratelli Cervi 93, Segrate (MI), 20054 Italy; 2 Institute of Agricultural Biology and Biotechnology, National Research Council (CNR), Via Edoardo Bassini 15, Milano, 20133 Italy; 3 Department of Animal Medicine, Health and Production, University of Padova, Viale dell’Universitá 16, Legnaro, 35020 Italy; 4 Department of Pharmaceutical and Pharmacological Sciences, University of Padova, Via Francesco Marzolo 5, Padova, 35131 Italy; 5 Department of Translational Medicine, University of Ferrara, Via Luigi Borsari 46, Ferrara, 44121 Italy

**Keywords:** Genomics, Archaea, Bacteria, Machine learning

## Abstract

**Background:**

Archaea and Bacteria are distinct domains of life that are adapted to a variety of ecological niches. Several genome-based methods have been developed for their accurate classification, yet many aspects of the specific genomic features that determine these differences are not fully understood. In this study, we used publicly available whole-genome sequences from bacteria ($N=2546$) and archaea ($N=109$). From these, a set of genomic features (nucleotide frequencies and proportions, coding sequences (CDS), non-coding, ribosomal and transfer RNA genes (ncRNA, rRNA, tRNA), Chargaff’s, topological entropy and Shannon’s entropy scores) was extracted and used as input data to develop machine learning models for the classification of archaea and bacteria.

**Results:**

The classification accuracy ranged from 0.993 (Random Forest) to 0.998 (Neural Networks). Over the four models, only 11 examples were misclassified, especially those belonging to the minority class (Archaea). From variable importance, tRNA topological and Shannon’s entropy, nucleotide frequencies in tRNA, rRNA and ncRNA, CDS, tRNA and rRNA Chargaff’s scores have emerged as the top discriminating factors. In particular, tRNA entropy (both topological and Shannon’s) was the most important genomic feature for classification, pointing at the complex interactions between the genetic code, tRNAs and the translational machinery.

**Conclusions:**

tRNA, rRNA and ncRNA genes emerged as the key genomic elements that underpin the classification of archaea and bacteria. In particular, higher nucleotide diversity was found in tRNA from bacteria compared to archaea. The analysis of the few classification errors reflects the complex phylogenetic relationships between bacteria, archaea and eukaryotes.

**Supplementary Information:**

The online version contains supplementary material available at 10.1186/s12864-024-10832-y.

## Background

In the complex and diverse realm of life, Archaea and Bacteria stand out as two fundamentally distinct domains within the kingdom Procaryotae, each characterized by their unique evolutionary trajectory and intrinsic properties [1]. The role of bacteria in ecosystems is pervasive, as witnessed also by the rapidly expanding research on microbiomes [2], while archaea have been traditionally viewed as marginal and associated predominantly with extreme habitats. The position of Archaea in the tree of life is now being re-evaluated in the light of new scientific discoveries, as recent advancements in genomics have significantly expanded our understanding of this domain. The archaeal family tree, once thought to encompass only two phyla, has blossomed into a more complex structure with the addition of new classes and superphyla, such as Methanonatronarchaeia, TACK, and the eukaryote-like Asgard group [3–5].

The advent of the $21^{\text {st}}$ century marked a significant development in the understanding of the microbial world, driven by advances in sequencing technologies (NGS, $3^{\text {rd}}$-generation sequencing) and computational methods [6]. Indeed, the inception of modern genomics can be traced back to the sequencing of the first bacterial genome in 1995 [7] and the first archaeal genome in 1996 [8]. These seminal events heralded an era of exponential growth in genomics, characterized by a doubling time for the number of available sequences of approximately 20 months for bacteria and 34 months for archaea [1]. This rapid accumulation of genomic data is reflected in the Reference Sequence Database (RefSeq: https://ftp.ncbi.nlm.nih.gov/genomes/refseq) of the National Center for Biotechnology Information (NCBI) which has meticulously documented $1\,222$ archaeal and $51\,425$ bacterial species. This collection encompasses annotated sequences of DNA, RNA and proteins which provide a standardized, reliable and publicly accessible set of reference sequences, and represent a pivotal resource in molecular biology and bioinformatics.

From the genetic point of view, archaea and bacteria display distinct biological processes regarding their DNA replication, transcription and translation machinery. While archaea share some similarities with eukaryotes in terms of transcription and replication, bacteria exhibit a different set of enzymes and pathways [9]. Metabolically, the two domains use varied pathways for energy production and biosynthesis, reflecting their adaptation to different ecological niches [10]. Also, regulatory elements such as transfer RNAs (tRNAs) have been a focus of recent research in understanding the evolutionary divergence between Archaea and Bacteria. Variations in tRNA patterns and their interaction with ribosomes provide insights into the evolutionary history and adaptation of these organisms [11].

From this perspective, the distinction between Archaea and Bacteria, which can often appear blurred at a first glance, becomes clearer under genomic scrutiny. While the analysis of the genomes of these organisms yields insights into their phylogeny [12], accurately identifying the specific genomic features that differentiate Archaea from Bacteria is still a challenge. This highlights the need of integrating advanced bioinformatics methods with high-throughput sequencing to enable more refined microbial classification and to uncover the subtle genomic distinctions between these domains. Thus, at the heart of current investigations is the utilization of modern machine learning (ML) algorithms for the analysis of genomic data. ML has already been applied to the study of prokaryotic genomes, e.g. to annotate archaeal promoters [13], to predict the evolution of bacterial metabolic systems [14], to understand complex anaerobic digestion mechanisms [15], to advance forensic microbiology [16], and much more (reviewed in [17, 18]). A recent work from our research group [19] exemplifies this approach, by applying ML algorithms to accurately differentiate between probiotic and non-probiotic microbial organisms, underlining the pivotal role of tRNAs for the accuracy of classification. These findings align with a growing evidence that places RNAs, especially tRNAs, in a central role for the modulation of gene expression and cellular regulation [20, 21].

Based on the above, it appears that it is of theoretical and practical interest to understand what drives the differentiation between archaea and bacteria at the genomic level. In this work we used multiple ML algorithms (Regularized Logistic Regression, Random Forest, Support Vector Machines and Neural Networks) to classify Bacteria and Archaea domains based on a set of genomic features (e.g. length in bps, proportion of coding and non-coding sequences, tRNA, rRNA and ncRNA genes). Given that we can expect the genomic classification of Archaea and Bacteria to be highly accurate, extracting variable importance allows us to identify the genomic features that underpin the differences between the two domains. A deeper understanding of the genomic elements that distinguish Archaea from Bacteria can provide insights into the evolutionary history of their genomes and how these elements have shaped their biological characteristics.

## Methods

### Dataset construction and encoding of genomic features

The dataset used in the present study included, after filtering, 2655 whole-genome sequences from bacteria (N=2546) and archaea (N=109) with good quality annotation. The GBRAP (GenBank Retrieving, Analyzing and Parsing) tool [22] was used to download genomic data from the NCBI GenBank [23] FTP databases for bacteria ( https://ftp.ncbi.nlm.nih.gov/genomes/refseq/bacteria/) and archaea (https://ftp.ncbi.nlm.nih.gov/genomes/refseq/archaea/). Only species with complete genomes were included. Contigs and scaffolds were directly excluded by GBRAP prior to the analysis. Bacterial genomes with size and number of coding sequences (CDS) smaller than that of *Mycoplasma genitalium* –now renamed *Mycoplasmoides genitalium*– ($< 580\,076$ bp and $< 491$ cds; [24]), and archaea genomes with size and number of CDS smaller than that of *Nanoarchaeum equitans* ($< 490\,885$ bp and $< 493$ cds; [25]) were removed. In addition, genomes lacking annotation in ribosomal, transfer and non-coding RNAs (rRNAs, tRNAs, and ncRNAs) were excluded. If more subspecies were present, the one with the longest genome was retained. The downloaded Genbank files of the selected microorganisms were used to calculate genomic statistics (e.g. nucleotide counts and relative frequencies, size in bps) on the whole genome and its components, i.e. CDS, rRNAs, tRNAs, ncRNAs. Briefly, the total number of bases (bp_total) for each element was obtained, together with the total count for each base (bp_A, bp_T, bp_C, bp_G) and their relative frequencies (fr_A, fr_T, fr_C, fr_G). The frequencies of each genomic component (CDS, rRNA, tRNA, ncRNA) on the plus strand (n_plus), on the minus strand (n_minus), and their sum (n_total) in the genome sequence were also calculated. In addition, the GBRAP tool calculated multiple genomic synthetic scores on the whole genome and subcomponents: Shannon’s entropy [26], topological entropy [27] and scores based on Chargaff’s second parity rule [28]. The Shannon’s and topological scores are associated with the concept of information entropy, which can be seen as the complexity of the message (e.g. the sequence ATGC has greater information content than AAAA). The two Chargaff’s scores refer to the deviation from the Chargaff’s second parity rule (details below). In summary, the dataset used in this study included the outcome to be predicted (the domain: Archaea or Bacteria) and 77 genomic features (57 genomic statistics and 20 scores) calculated for each of the 2655 microorganisms considered (109 archaea and 2546 bacteria). The 77 genomic features, which were in part previously described in Bergamini et al. (2022 [19]), are listed in S1 Table.

### Genomic scores

The four genomic scores described below were calculated from GBRAP on the microbial sequences downloaded from the NCBI repository. The scores were calculated on: the whole-genome, CDS, rRNA, tRNA, and ncRNA, for a total of 20 genomic score features to be used in the predictive models.

#### Shannon’s entropy

Shannon’s entropy (or information entropy [26]) is used in genomics to quantify the uncertainty or complexity in a set of sequences. It serves as a measure of the randomness or variability in genetic sequence data. The Shannon’s entropy (H) of a sequence is formally calculated as:

1 $$\begin{aligned} H = - \sum (p_i \cdot log2(p_i)) \end{aligned}$$

where $p_i$ represents the proportion of each nucleotide or amino acid in the sequence.

#### Topological entropy

Topological entropy is a theoretical measure that quantifies the complexity or degree of randomness within infinite sequences. Differently from Shannon’s entropy, which evaluates the uncertainty or information content within a finite probabilistic distribution of events or symbols, topological entropy focuses on the asymptotic exponential rate of distinct substrings as the length of the sequence increases. Topological entropy was therefore introduced to study the complexity of infinite sequences, making its direct application to finite sequences challenging due to limited sampling effects and high-dimensionality issues.

Koslicki et al. (2011 [27]) introduced a new approximation of topological entropy that overcomes these difficulties, making it applicable to finite length sequences like DNA, while retaining connections with information theory. This new definition enables the comparison of entropy between sequences of different length, a property not incorporated in previous implementations of topological entropy.

Topological entropy in genomics is calculated based on the diversity of short repeated sub-sequences within a DNA sequence. Essentially, it counts how many different patterns of given length appear in the sequence. A higher variety of patterns indicates higher entropy, suggesting a more complex and less repetitive DNA region.

#### Chargaff’s scores

Chargaff’s score is used to assess a genome’s adherence to Chargaff’s second parity rule. This rule states that, in double-helical DNA the amount of adenine (A) and thymine (T) is approximately equal to that of cytosine (C) and guanine (G) on each single strand (with the exception of mitochondrial DNA). The Chargaff’s score can be calculated with two methods:

1. The PF method uses the AT and GC skews:
  $$\text {AT skew} = \frac{|(\#A - \#T)}{(\#A + \#T)|}$$ 
  $$\text {GC skew} = \frac{|(\#C - \#G)}{(\#C + \#G)|}$$ 
    where # indicates the frequency (counts) of the four nucleotides. The sum of the two skews quantifies the deviation from perfect parity (a score of 0 indicates perfect adherence to Chargaff’s rule).
2. The CT method (from GBRAP [22]) calculates the score as the average of the A/T and C/G ratios, where the least frequent nucleotide is chosen as the numerator:
  $$\text {Chargaff's score} = 0.5 \cdot \left( \frac{min(\#A, \#T)}{max(\#A, \#T)} + \frac{min(\#C, \#G)}{max(\#C, \#G)} \right)$$ 

- This version of the score ranges from 0 to 1, where 1 represents perfect Chargaff’s parity.

Chargaff’s score is widely used in genomics as an indicator of genome stability, as a score approaching perfect parity suggests a stable genomic structure [28]. The two ways of calculating the Chargaff’s score have their own peculiarities (different ranges, different sensitivity to bias linked to sequence length), and in this study they provide two different genomic features to be used as input by ML predictive models.

### Exploratory data analysis (EDA)

As data exploration steps, we used the 77 genomic features extracted from GBRAP for Principal Component Analysis (PCA), correlation analysis, and clustering. In PCA, we assessed how well the data could be compressed into a reduced number of variables (principal components: PCs), and how much of the total variance of the data was accounted for by PCs, by looking at the ratio of the corresponding eigenvalues over the sum of all eigenvalues. For correlation analysis, the Pearson linear correlations between all pairs of genomic features were calculated. For the clustering of the 2655 microorganisms, we first calculated their pairwise Euclidean distances based on the genomic features, and then used multidimensional scaling to plot the resulting distance matrix.

### Model building, training and evaluation

**Fig. 1:**
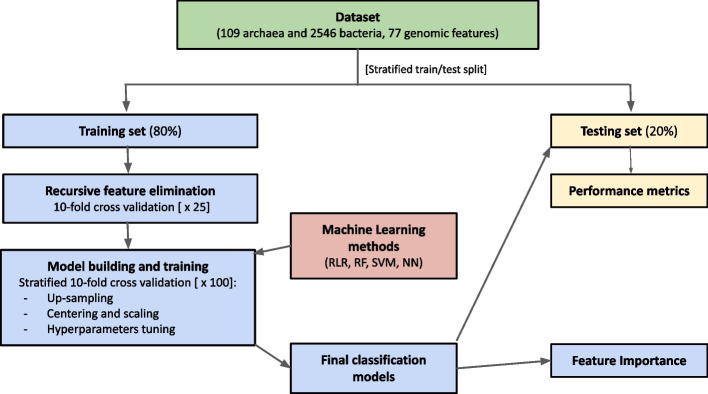
ML workflow. Machine learning workflow for the prediction of the taxonomic domain (Bacteria or Archaea) using genomic features. RLR: regularized logistic regression; RF: Random Forest; SVM: support vector machines; NN: neural networks

The analysis workflow is summarized in Fig. 1 and was executed with the Caret v.6.0-86 [29] and Tidyverse v.1.3.1 [30] R packages (R v.4.1.2 [31]). First, a subset with 80% of the records (88 archaea and 2037 bacteria) was sampled to build and train the predictive models; the remaining 20% of the data (21 archaea and 509 bacteria) was excluded from model building and used as test set (unseen labels) to evaluate model performance in discriminating between the two taxonomic domains. Stratification was applied at subsetting to preserve the original class distribution (4.1% archaea and 95.9% bacteria) in both the training and test sets.

Before model building, automatic backward selection of features was performed on the training set, applying the recursive feature elimination (RFE) algorithm based on random forest (RF) [32]. RFE was based on the average accuracy of prediction from 10-fold cross-validation (CV) repeated 25 times. The rationale of applying RFE before model building is to identify the most predictive features to be included in the most parsimonious model reaching the greatest accuracy of prediction. In particular, for each resampling iteration, training data are further partitioned into training and validation sets. The algorithm fits the RF model on the training set using all features, which are ranked according to their predictive importance on the validation set, and the less important ones are sequentially eliminated. Indeed, for each feature subset to be tested, rankings are re-computed, until the appropriate number of features is determined. The goal is to find the minimum set of data needed for accurate predictions (see Biscarini et al. 2015 [33] for an example).

Retained genomic features were then used to predict the taxonomic domain of the microorganisms (binary classification problem: Bacteria or Archaea) applying four ML methods: i) regularized logistic regression (RLR) with Ridge, Lasso or Elastic-Net penalties [34]; ii) random forest with 500 classification trees (RF [35]); iii) Support Vector Machines with radial basis function (RBF) kernel (SVM [36]), and iv) a one-layer neural networks model: one hidden layer, one output node, sigmoid activation function at every node (NN [37]). The following hyperparameters were fine-tuned: i) the type ($\alpha$: Ridge, Lasso, Elastic-Net) and amount ($\lambda$) of penalization for the RLR models; ii) the number of features randomly sampled to be used in each classification tree of the RF models; iii) the amount of flexibility (cost “C”) for the SVM classifier; iv) the number of units in the hidden layer and the L2 penalty (weight decay) to apply to the estimated coefficients for the NN models.

Training and validation of the models was performed applying a stratified (by domain) 10-fold CV repeated 100 times. Briefly, the training set was divided into 10 subsets of equal size, nine used for model training and one for validation. The entire process was repeated 100 times. Therefore, 100 mean accuracy values were then averaged to obtain the final metrics of each method to be compared. To resolve class imbalance of the domains, additional up-sampling was conducted inside the resampling, i.e. randomly sampling (with replacement) the low-frequency class to be the same size as the high-frequency class.

Data preprocessing (centering and scaling) was done within CV, after up-sampling. For each tested method, the train function of the Caret R package automatically created a grid of tuning hyperparameters. By default, the grid size is $3^p$, where *p* is the number of tuning hyperparameters specific to each method. The combination of hyperparameter values corresponding to the best performance (accuracy) was then chosen as the final model to be fitted on the training set. Tuning details of the hyperparameters of each ML method are reported in the S1 Appendix.

### Metrics for the evaluation of model performance

For this binary classification problem, Bacteria (the majority class) were considered “positive” cases while Archaea (the minority class) were considered “negative” cases. The performance of the four ML methods on the validation set (fine-tuning of the hyperparameters through CV) was evaluated in terms of classification accuracy (proportion of correctly classified observations). The metric was calculated as the ratio between correct predictions over all predictions:

2 $$\begin{aligned} \text {accuracy} = \frac{\text {TP} + \text {TN}}{\text {TP}+\text {TN}+\text {FP}+\text {FN}} \end{aligned}$$

where TP is the number of true positives, TN that of true negatives, FP and FN are false positives and false negatives. The model with the greatest values of accuracy was then used to rank the importance of genomic features in predicting the taxonomic domain.

The predictive ability of the ML methods on the test set (final evaluation of model performance) was assessed based on several metrics obtained from the confusion matrix: accuracy, true positive rate (TPR), true negative rate (TNR), positive predictive value (PPV), negative predictive value (NPV), the Matthew’s Correlation Coefficient (MCC). For clarity, MCC ranges in [-1,1] and was calculated as:

3 $$\begin{aligned} \text {MCC} = \frac{ (\text {TP} \cdot \text {TN}) - (\text {FP} \cdot \text {FN}) }{ \sqrt{ (\text {TP}+\text {FP}) \cdot (\text {TP}+\text {FN}) \cdot (\text {TN}+\text {FP}) \cdot (\text {TN}+\text {FN})} } \end{aligned}$$

## Results

### Feature selection and model building

**Fig. 2:**
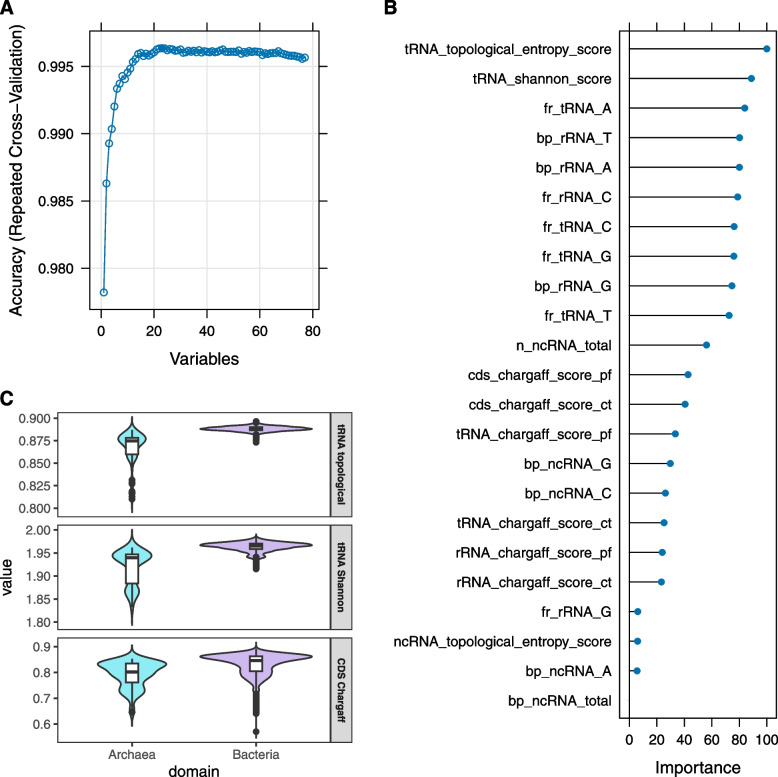
Variable importance. **A**) Results of the recursive feature elimination, based on Random Forest. The number of features included in the model and the prediction accuracy are reported on the x-axis and on the y-axis, respectively; **B**) Plot of the 23 most important features for the prediction of the Bacteria/Archaea domain, using Support Vector Machine as the best predictive method. These are all features identified by the model during RFE (recursive-feature elimination). Importance values have been rescaled in 0-100; **C**) Comparative analysis of tRNA topological entropy, tRNA Shannon’s entropy and CDS Chargaff’s Score CT across archaea and bacteria. The violin plot, augmented with internal boxplots, displays the distribution and median values of the three genomic features for both Archaea (in light blue) and Bacteria (in light purple). The facets separately highlight each variable, providing insights into the genomic distinctions between these two domains

The genomic features used to develop the four ML predictive models were obtained from the GBRAP tool, which allowed the calculation of several DNA and RNA-genes properties, including genome size, number and frequency of base pairs, and scores related to the genomic information content. S1 Fig. reports results from EDA: i) scree plot of the PCs in decreasing order of the percentage of variance they explained (the first three PCs accounted for $61.5\%$ of the total variability in the data); ii) correlation plot of the genomic features; and iii) multidimensional scaling plot of Euclidean distances between microorganisms (bacteria, archaea) based on the matrix of genomic features. Before model building and training, RF-based RFE was applied to remove least informative features and minimize the set of data needed to reach the greatest possible predictive ability. Using resampling (10-fold CV with 25 repeats), multiple lists of predictors to be retained were generated from which a consensus ranking was obtained. This approach provides a more reliable assessment of feature importance in comparison to a single fixed ranking. The best subset was found to be that with 23 predictors (Fig. 2A): tRNA_topological_entropy_score, tRNA_shannon_score, fr_tRNA_A, fr_rRNA_C, ncRNA_topological_entropy_score, cds_chargaff_score_pf, rRNA_chargaff_score_pf, cds_chargaff_score_ct, fr_tRNA_C, n_ncRNA_total, bp_ncRNA_G, rRNA_chargaff_score_ct, bp_ncRNA_total, bp_ncRNA_C, tRNA_chargaff_score_pf, bp_rRNA_T, fr_tRNA_G, bp_rRNA_G, fr_tRNA_T, tRNA_chargaff_score_ct, bp_ncRNA_A, bp_rRNA_A, fr_rRNA_G.

### Final model and feature importance from cross-validation

The evaluation of the predictive performance of the four ML methods on the validation set (from cross-validation for fine-tuning of the hyperparameters) was based on the overall accuracy (Table 1). All algorithms reached a prediction accuracy $> 99\%$, from $99.7183\%$ for RLR and RF to $99.9531\%$ for SVM.

Features were ranked by importance from SVM, the best predictive method after cross-validation: tRNA topological entropy, tRNA Shannon’s entropy score, nucleotide absolute (bp) and relative (fr) frequencies in tRNAs and rRNA, total frequency of ncRNA and CDS Chargaff’s score (both PF and CT methods) were found to be the most important features for domain prediction on the validation set (Fig. 2B). For all features, values were significantly different in bacteria compared to archaea (Wilcoxon test: *p*-value $<< 0.01$, S2 Table). Notably, seven and eight out of 23 features are related to the nucleotide composition, entropy, and stability of rRNAs and tRNAs, respectively.

**Table 1:** Model performance. Accuracy on the validation set from 10-fold cross-validation (CV: fine-tuning of the hyperparameters). Accuracy, true positive rate (TPR), true negative rate (TNR), positive predictive value (PPV), negative predictive value (NPV) and Matthew’s Correlation Coefficient (MCC) on the test set

Method	10-fold CV (validation set)	Model performance (test set)					
	Accuracy	Accuracy	TPR	TNR	PPV	NPV	MCC
Regularized logistic regression	0.997183	0.994	0.996	0.952	0.998	0.909	0.928
Random Forest	0.997183	0.993	0.998	0.857	0.994	0.947	0.897
Support vector machines	0.999531	0.994	0.998	0.905	0.996	0.950	0.924
Neural networks	0.999528	0.998	1.0	0.952	0.998	1.0	0.975

For model training, 80% of the data were used: 10-fold cross-validation (repeated 100 times) was used within the training set for the tuning of the hyperparameters. For testing, 20% of the data were left aside and used for the evaluation of the final models

### Predictive performance on the test set

The predictive performance of the four final ML models after cross-validation was evaluated on the test set (20% of the initial data) based on summary metrics (Table 1) and on the confusion matrix (error breakdown, Table 2). NN was found to be the best model to predict the domain based on genomic features, with an accuracy of prediction of $99.8\%$ and the best agreement between the predicted and actual values (MCC = 0.975). NN was followed by RLR, SVM and RF in terms of predictive ability of the model.

**Fig. 3:**
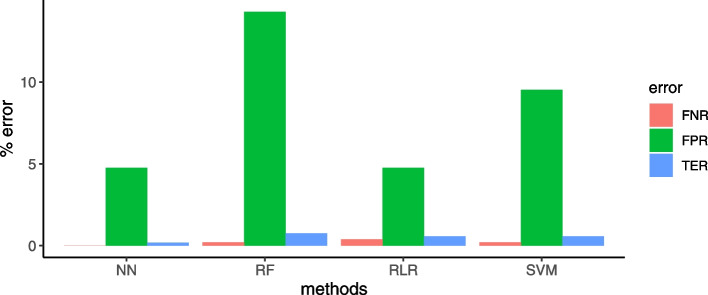
Error rates. False negative error rate (FNR), false positive error rate (FPR) and total error rate (TER) for the prediction of the Archaea ( = Negative) or Bacteria ( = Positive) domain on the test set of four machine learning methods: Regularized logistic regression (RLR), Random Forest (RF), Support vector machines (SVM), and Neural networks (NN)

Taking Archaea as “negatives” and Bacteria as “positives” in the binary classification problem, our results show: 0.40% false negative error rate (FNR) for RLR, and 0.2% FNR for both RF and SVM; 4.76% false positive error rate (FPR) for both NN and RLR, 9.5% FPR for SVM and 14.3% FPR for RF (Fig. 3). In particular, the archaea *Methanobrevibacter ruminantium* M1 was misclassified as bacterium by all four classification models, with $P(y=1|x) \in [0.87,1]$. The bacterium *Deferribacter desulfuricans* SSM1 was misclassified as archaea by both RF ($P(y=0|x) = 0.78$) and SVM ($P(y=0|x)=0.55$). RLR misclassified 2 out of 509 bacteria as Archaea (*Gottschalkia acidurici 9a*, *Tautonia plasticadhaeren*). All other mistakes were made by only one classifier. Details on the misclassifications are reported in Table 3.

**Table 2:** Confusion matrix. Confusion matrices for the prediction of the Archaea/Bacteria domain on the test set of four machine learning methods: Regularized logistic regression (RLR), Random Forest (RF), Support vector machines (SVM), and Neural networks (NN)

		Reference	
Method	Predictions	Archaea	Bacteria
RLR	Archaea	20	2
	Bacteria	1	507
RF	Archaea	18	1
	Bacteria	3	508
SVM	Archaea	19	1
	Bacteria	2	508
NN	Archaea	20	0
	Bacteria	1	509

## Discussion

In this paper, we explored the application of ML methods to analyze genomic data for the classification of microbial samples belonging to the life domains Bacteria and Archaea. ML has already been extensively applied to the study of microbial populations, and has proven instrumental in forecasting disease conditions, evaluating environmental integrity, detecting the presence of contaminants in ecosystems and in forensic investigations [17]. Our main objective was to develop ML predictive models and, based on the expected high accuracy of prediction, extract the genomic features that are important for prediction and are hence helpful to differentiate microorganisms belonging to either the Bacteria or Archaea domain. We discuss hereby the identified genomic features, the obtained accuracy of prediction with a detailed analysis of the few classification errors, and the biomedical and biotechnological implications of these results.

### Feature importance: What drives the genetic differences between archaea and bacteria

As expected, the ML models that we tested were all able to distinguish with very high accuracy between samples from the domains Archaea and Bacteria, based on their genomic data. The key drivers of this highly accurate classification were the entropy of tRNAs (tRNA topological entropy, tRNA Shannon’s entropy score), the nucleotide absolute (bp) and relative (fr) frequencies in tRNAs and rRNA, and Chargaff’s scores -with both the PF and CT methods- of CDS. Interestingly, 7 out of the total 23 important variables identified by RFE are related to rRNA (nucleotide frequencies, Chargaff’s scores). This result highlights the significant role of rRNA genes in phylogenetic studies of bacteria and archaea, as shown also by the common use of rRNA marker genes to study the composition of microbial communities, which is at the foundation of the entire scientific field of metataxonomics (e.g. 16S/18S/23S rRNA-gene sequencing [38, 39]).

Focussing on the top two features from Fig. 2B, while tRNAs are predominantly recognized for their role in protein synthesis, their broader impact on gene expression regulation and cellular processes has been increasingly acknowledged [40, 41]. This multifunctionality makes them a critical molecular component to understand the genomic differentiation between the two domains (Archaea *vs* Bacteria). It is worth mentioning that the complexity of tRNA functions extends beyond prokaryotes to oncogenesis in humans and mammals, where expression, modifications and aberrations of tRNA molecules are linked with cancer development [42, 43]. The observed higher topological and Shannon’s entropy in bacterial tRNAs (Fig. 2C) may be reflective of their adaptation to diverse ecological niches. Further dissecting the reasons behind the higher entropy in bacterial tRNAs, a number of hypothetical explanations may be formulated:

- Rapid Evolution and Mutation Rates (REMR): bacteria exhibit faster reproduction rates compared to archaea and eukaryotes, potentially leading to quicker evolutionary adaptations. This rapid evolution could result in increased mutation rates in various genes, including those encoding tRNAs, thus contributing to higher entropy [44];
- Environmental Niche Diversity (END): bacteria have the ability to thrive in a wide range of environments from extreme conditions to various biomes within the human body, and might necessitate a diverse set of tRNA gene sequences. This diversity aids in adapting protein synthesis to different environmental conditions, which would increase the entropy of their tRNA genes [45];
- Genetic Code Plasticity (GCP): the significant plasticity observed in the bacterial genetic code, which includes variations to the standard genetic code, may extend to tRNA genes. This flexibility could contribute to the diversity and complexity of tRNA sequences, increasing their entropy [46, 47];
- Translation Needs (TN): although also archaea experience varying environmental conditions, the larger number of diverse environments in which bacteria are found might justify a broader array of tRNA molecules to meet distinct translation demands. This need for diverse tRNA sets could lead to higher entropy in their tRNA genes [48];
- Horizontal Gene Transfer (HGT): the potential for HGT in bacteria to introduce new genetic material, such as tRNA genes from diverse organisms, can significantly enhance the diversity of the bacterial tRNA gene pool, contributing to the observed higher entropy. Although HGT occurs in archaea as well, there are notable differences in HGT frequencies across different bacterial and archaeal taxa [49]. These differences may impact genomic diversity and complexity in distinct ways within each domain, which could help explain the variability observed in our analysis.

It is intriguing to observe that bacterial CDS exhibit more extreme Chargaff’s scores (higher Chargaff-CT score, lower Chargaff-PF score), indicating overall greater sequence stability compared to archaea. This finding initially appears counterintuitive, considering the prevalence of archaea in extreme environments where genomic stability would presumably be a crucial adaptive trait. Nevertheless, we noticed that the median CDS Chargaff-CT score in archaea (0.80) lies between that of bacteria (greater than 0.85) and eukaryotes (0.77: based on 24 high-quality eukaryotic genomes from NCBI, including mammals, birds, fish, reptiles, amphibians, invertebrates, fungi and plants; data not shown). This observation suggests that as CDS become less topologically random during evolution, the strict applicability of Chargaff’s second parity rule diminishes, especially within coding genomic regions subjected to evolutionary pressure towards conservation. Koslicki (2011, [27]) demonstrated that human introns exhibit higher topological entropy compared to exons, indicating greater randomness. Similarly, our results show that the topological entropy of archaeal CDS is intermediate between that of bacterial and eukaryotic CDS (S2 Fig.), mirroring the pattern observed with Chargaff-CT scores. This suggests that prokaryotic CDS have higher topological randomness than eukaryotic CDS. Archaea plausibly represent an evolutionary intermediate, placed between these two extremes. Alternatively, it is also possible that the difference in Chargaff’s score can be influenced by other factors such as different mechanisms of DNA repair, replication fidelity, or even variations in HGT processes between the two domains.

### Classification accuracy and error analysis

The accuracy of classification achieved by the four ML models ranged between 0.993 (RF) and 0.998 (NN). Many approaches have been developed for the classification of bacteria and archaea [50], and genomic-based approaches stand out as being highly accurate. The dataset analysed in this work was imbalanced, with 2546 bacteria (considered the ‘positive’ cases) and 109 archaea (‘negative’ cases), and the few classification errors therefore tended to be more frequently false positives (Archaea misclassified as Bacteria) than false negatives (Bacteria misclassified as Archaea). Here we compare the performance of the four ML models given the imbalanced classes, and then analyse in detail the few classification errors that have been obtained.

#### Imbalanced classes: Area under the ROC curve (AUC) vs MCC

In this work, we compared the relative predictive performance of four ML models in a binary classification problem using several metrics, particularly error ratios from the confusion matrix and MCC. Considering multiple performance metrics is a common approach to model evaluation, since it may be relevant to look not only at the total errors but also at the errors in the two classes. This is especially important when the dataset is imbalanced, with many examples of one class and few of the other: in such cases, the accuracy (Eq. 2) may provide an inflated and overly optimistic view of model performance [51]. The area under the ROC (receiver operating characteristic) curve (AUC) is a widely used metric for the evaluation of classification models on balanced and imbalanced binary prediction problems that combines the accuracy in both classes: AUC summarizes results over all possible classification thresholds, removes as a consequence the subjectivity of choosing a threshold, and makes a trade-off between TPR and FPR (thereby avoiding models that look deceptively good by predicting well in the majority class) [52]. Yet, to compare our four models we did not use AUC: the reason is that AUC has drawbacks and in some cases can be a misleading measure of the model performance. AUC only takes into account TPR and FPR = 1-TNR (the accuracy measured on the true labels), but when data are strongly imbalanced this can be suboptimal: if, for instance, the number of positive examples (one class) greatly exceeds the number of negative examples (the other class), a sizeable change in the number of false negatives can lead to a small change in the false negative rate used in the calculations of AUC. This is exactly what happened in our work: the most false negative errors were made by RLR (FN = 2: “Bacteria” –the “positives”, in the convention used here– misclassified as Archaea). Given the large number of Bacteria in our dataset (2546, 95.9% of the data), two Bacteria predicted as Archaea translates to TPR = 1-FNR = 0.996 and FPR = 0.048 (the two rates used by AUC). Conversely, three of the four errors made by RF were in the “Archaea” class (“negatives”, in the convention used here), which on one hand leads to TPR = 0.998, but on the other gives FPR = 0.143. The AUC calculated on the results from the classification models tested in this work were 0.998 for RLR, 0.997 for RF, 1 for SVM, and 0.976 for NN (see S3 Fig.). Clearly, the results from AUC would be misleading in this case, giving NN, the model that gave the best results (only one error), a worse performance than RF, the model that made the most errors. This shortcoming of AUC under extreme conditions is known in literature [53, 54], together with the risk of misusing this metric [55]. Positive predictive value (PPV, a.k.a. precision), on the other hand, by comparing false positives to true positives rather than true negatives, captures the effect of the large number of negative examples on the algorithm’s performance. The same is true, *mutatis mutandis*, for the negative predictive value (NPV). From Table 1, we see that looking at ratios over predictions, the relatively poor performance of RLR in the Archaea class is highlighted (NPV = 0.909). This is why to evaluate our results we decided to use the confusion matrix and MCC: the confusion matrix gives the overall breakdown of errors, while MCC combines all four rates (TPR, TNR, PPV, NPV), thereby giving a complete picture of the model’s predictive ability. MCC values are high only if the classifier gave high values for all the four accuracy rates. Additionally, a high MCC value always corresponds to a high ROC AUC, while a given TPR-TNR pair can cover a broad MCC range [56]. Another approach to the evaluation of binary classifiers is represented by cost curves [57], which factor in not only the frequency of the classes but also the relevance (cost) of the different types of mistakes. This is not applicable, though, to problems where mistakes in the two classes are equivalent (they have the same cost), i.e. there is no more dangerous or more important class, as is the case of our Archaea and Bacteria genomic classification.

**Table 3:** Error analysis. Detailed view of the 11 classification errors made cumulatively by the four predictive models tested in this work: RLR (Regularised Linear Regression), RF (Random Forest), SVM (Support Vector Machine), NN (Neural Networks)

GenBank ID	Genus, species	Misclassified by	Probability	Prediction	Domain	Clade	Phylum	Class	Order	Family
NC_013939	*Deferribacter desulfuricans* SSM1	RF, SVM	0.78, 0.55	Archaea	Bacteria	-	*Deferribacterota*	*Deferribacteres*	*Deferribacterales*	*Deferribacteraceae*
NC_018664	*Gottschalkia acidurici* 9a	RLR	0.53	Archaea	Bacteria	Terrabacteria group	*Bacillota*	*Tissierellia*	*Tissierellales*	*Gottschalkiaceae*
NZ_CP036426	*Tautonia plasticadhaerens*	RLR	0.98	Archaea	Bacteria	PVC group	*Planctomycetota*	*Planctomycetia*	*Isosphaerales*	*Isosphaeraceae*
NZ_CP012850	*Candidatus Nitrosocosmicus oleophilus*	SVM	0.94	Bacteria	Archaea	TACK group	*Nitrososphaerota*	*Nitrososphaeria*	*Nitrososphaerales*	*Nitrososphaeraceae*
NC_013790	*Methanobrevibacter ruminantium* M1	RLR, RF, SVM, NN	0.97, 0.99, 0.87, 1	Bacteria	Archaea	Methanomada group	*Euryarchaeota*	*Methanobacteria*	*Methanobacteriales*	*Methanobacteriaceae*
NZ_CM001555	*Methanofollis liminatans* DSM 4140	RF	0.73	Bacteria	Archaea	Stenosarchaea group	*Euryarchaeota*	*Methanomicrobia*	*Methanomicrobiales*	*Methanomicrobiaceae*
NC_007796	*Methanospirillum hungatei* JF-1	RF	0.93	Bacteria	Archaea	Stenosarchaea group	*Euryarchaeota*	*Methanomicrobia*	*Methanomicrobiales*	*Methanospirillaceae*

The taxonomy (Domain, Clade, Phylum, Class, Order, Family) was retrieved from the NCBI Taxonomy browser (https://www.ncbi.nlm.nih.gov/taxonomy)

#### Error analysis

From Table 2, we see that over all four classification models 11 examples were misclassified in total, 3 bacteria (one twice) and 4 archaea (one four times): these are detailed in Table 3. In particular, the archaea *Methanobrevibacter ruminantium* M1 has been misclassified by all four models, with probabilities ranging from 0.87 (SVM) to 1 (NN). The erroneous classification of this archaea can be explained by its high values of tRNA topological entropy (0.8868) and tRNA Shannon’s entropy (1.9616), which are closer to the median values observed for bacteria (0.888 and 1.97) rather than to those of archaea (0.875 and 1.94, Fig. 2C).

**Fig. 4:**
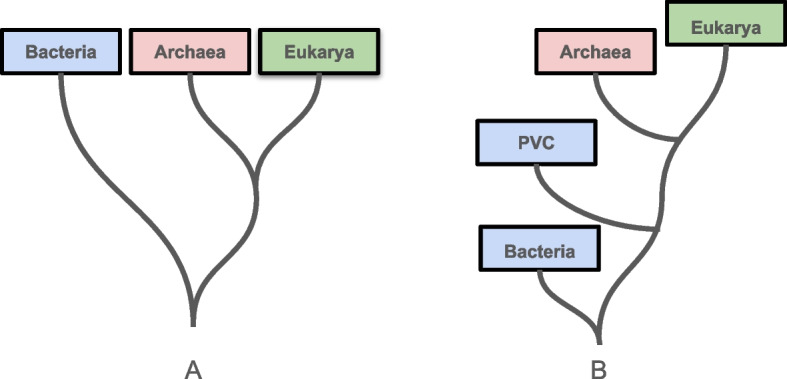
The tree of life. **A** The traditional three-domains tree of life and (**B**) the PVC-based one-domain tree of life (Devos et al., 2021)

Similarly, the bacteria *Tautonia plasticadhaeren*, misclassified as archaea by RLR with $p(x)=0.98$, has tRNA topological entropy and tRNA Shannon’s entropy of 0.8732 and 1.9202, even lower than the median values found in archaea. *Tautonia plasticadhaeren* belongs to the PVC superphylum. This group includes the phyla *Planctomycetes*, *Verrucomicrobia* and *Chlamydiae* (founders of the PVC group), as well as *Lentisphaerae*, *Kirimatiellaeota* and some uncultured candidate phyla [58]. Results from transcriptomics confirmed the presence of genes associated with eukaryotic cellular functions, like membrane fusion, that might have enabled PVC microorganisms to evolve features typical of eukaryotic cells [59]. Indeed, some members of this superphylum present cellular features typical of archaea or eukaryotes, e.g. actin/tubulin-based microfilaments, endoplasmic reticulum, Golgi apparatus, vacuoles and vesicles [60]. The presence of eukaryotic signatures might explain the misclassification of a PVC bacterium as Archaea. Our findings reflect the complex and ever-changing evolutionary relationship between prokaryotes and eukaryotes [61]. Numerous phylogenetic analyses of archaeal and bacterial genomes, such as the work on Asgard archaea [62, 63], provide stronger evidence for the relationships among the three domains of life. However, it is interesting to note that our ML analysis identified a PVC as an outlier, which recalls earlier theories about their distinct evolutionary trajectories. These results add a complementary perspective to the ongoing exploration of these evolutionary relationships, recalling the hypothesis of a PVC-based eukaryogenesis process, where the PVC bacteria ancestor diverged by developing features typical of archaea and eukaryotes [59, 60]. Actually, the discovery of Archaea initially led to hypothesize a tree of life composed of three domains characterized by independent origins, with Archaea positioned closer to Eukarya (Fig. 4A [64]). Advancements in phylogenetics highlighted possible alternative scenarios, including the hypothesis formulated by Devos et al. [60] of a PVC-based single bacterial ancestral domain (Fig. 4B).

The exponential increase in the annual tally of sequenced microbial genomes permits to hypothesize a closer relationship between Archaea and Eukarya as compared to Bacteria, challenging conventional perspectives and underscoring a complex evolutionary history [64–66]; yet, deciphering the relationships between life domains is still a difficult and partly unresolved topic.

### Biomedical and biotechnological implications

Our study provides a set of genomic features and ML predictive models for discriminating microorganisms belonging to either the Bacteria or Archaea domain. While recognizing the value of taxonomic classification tools and repositories (e.g. Genome Taxonomy Database (GTDB: [67]), NCBI Taxonomy [68]), which provide extensive phylogenetic analysis, our approach is different as it focuses on a broader comparison of different taxa using unique genomic features such as Chargaff’s score, topological entropy, and Shannon’s entropy across various genomic elements including total genome, CDS, rRNA, tRNA, and ncRNA. These features are currently not available in other software packages or public repositories, which highlights the unique value of the present work in complementing existing phylogenetic resources. Furthermore, the results of this study can be helpful in phylogenomics, providing potential additional marker genes to be used in metataxonomics (e.g. tRNA, ncRNA genes) [69, 70]. The approach presented in this work, with appropriate modifications, could be geared towards addressing different classification problems. For instance, in a previous study [19] we demonstrated how ML algorithms and genomic data can identify novel probiotics, beneficial symbionts of the human gut microbiome, and also discriminate them from pathogenic organisms. This novel methodology can open new frontiers in biomedical research, enabling the monitoring of microbial dysbiosis involved in a wide range of disorders such as cancer, autoimmune and chronic intestinal diseases [71]. Additional biomedically relevant classification problems that can potentially be addressed, are for example the identification of beneficial bacteria, like Lactobacillus [72] and Bifidobacterium [73] that can be used to counteract pathogens such as *Helicobacter pylori* [74] (risk factor for ulcers and gastric cancer), or *Clostridioides difficile* (which can cause severe post-antibiotic infections [72]).

On the technological side, coupling ML techniques and a reduced set of microbial features extracted from the genome sequence improved the discovery rate of novel microorganisms with antifungal activity against plant pathogens [75]. ML approaches were recently adopted to predict the evolution of metabolic systems in bacteria [14], discovering evolutionary patterns which can potentially affect different biological fields (e.g., genome editing, pathogen control, synthetic biology). Tools based on ML methods applied to genomic sequences were developed for host prediction of viruses infecting bacteria and archaea [76], aiming at supporting the characterization of uncultivated viruses. Uncultured archaea from the environment, which can produce different cellular components with valuable applications on both green energy production and medicine, can be classified with good accuracy using ML models [77]. Recently, ML approaches have been coupled with environmental DNA (eDNA) to explore biological diversity of ecosystems and to provide novel insights about uncharacterized taxa [78, 79]. A future step would be the implementation of our approach also to not-completely annotated genomes, including unassembled contigs and scaffolds. The integration of artificial intelligence (AI) and microbiomics provides a substantial advancement in forensic science, for the identification and classification of microorganisms, as well as for a deeper understanding of the human post-mortem microbiome [16]. Considering that most of the microbial genomes have not been sequenced yet, the molecular functions of several genes are unknown and that many proteins have not been functionally annotated, the application of AI could illuminate the “microbial dark matter” of life [80].

## Conclusion

In this study, leveraging ML techniques applied to genomic data, we classified microorganisms belonging to the life domains Bacteria and Archaea, and discerned unique genomic discriminators between them. The higher sequence entropy in bacteria may suggest their need for more dynamic and versatile genetic configurations at the tRNA level. This is likely influenced by the diverse environmental niches they inhabit, demanding greater genomic plasticity and adaptability. Our results add to the existing knowledge on tRNA biology, emphasizing that these molecules are not mere bystanders in cellular dynamics. They are pivotal players, acting as conduits between genetic information and functional cellular activities. Understanding the nuanced differences in tRNA characteristics between Archaea and Bacteria offers a deeper insight into the biology of these two foundational domains of life and paves the way for further studies in other taxa. In addition, the analysis of the classification errors observed in the present study reflects the complicated phylogenetic relationships between bacteria, archaea and eukaryotes.

## Supplementary information

Supplementary file 1.

Supplementary file 2.

Supplementary file 3.

Supplementary file 4.

Supplementary file 5.

Supplementary file 6.

## Data Availability

Genomic data were downloaded from the NCBI GenBank FTP databases for bacteria (https://ftp.ncbi.nlm.nih.gov/genomes/refseq/bacteria/) and archaea (https://ftp.ncbi.nlm.nih.gov/genomes/refseq/archaea/). The specific dataset used for this analysis, after applying the filtering criteria, can be found in the following public repository: https://zenodo.org/records/13235119.

## Code availability

The R code used to develop the four classifier models for the analysis of the data in this work can be found in the following Github repository: https://github.com/filippob/ml_classification/blob/main/scripts_for_papers/archaea_classifiers_BMC.R.
