# Supplementary material for: Machine learning classification of archaea and bacteria identifies novel predictive genomic features
Source: BMC Genomics. 2024 Oct 14;25:955. doi: 10.1186/s12864-024-10832-y (PMC11472548; doi:10.1186/s12864-024-10832-y)
Supplement: Supplementary file 1 [file 12864_2024_10832_MOESM1_ESM.pdf]

S1 Table. List of the genomic features used in the study.

Feature	Description
Outcome	organism domain belonging (bacteria or archaea)
ID	GenBank identification code
bp_genA	total number of Adenines in the genomic sequence
bp_genT	total number of Thymines in the genomic sequence
bp_genC	total number of Cytosines in the genomic sequence
bp_genG	total number of Guanines in the genomic sequence
bp_genome_total	total number of bases in the genomic sequence
fr_genA	frequency of Adenine nucleotides in the genomic sequence
fr_genT	frequency of Thymine nucleotides in the genomic sequence
fr_genC	frequency of Cytosine nucleotides in the genomic sequence
fr_genG	frequency of Guanine nucleotides in the genomic sequence
genomic_topological_entropy_score	Topological score calculated on genomic sequences
genomic_chargaff_score_pf	Chargaff's second parity rule score calculated on genomic sequences using PF method
genomic_chargaff_score_ct	Chargaff’s second parity rule score calculated on genomic sequences using CT method
genomic_shannon_score	Shannon’s score calculated on genomic sequences
n_cds_plus	number of coding sequences on the + strand of the genomic sequence
n_cds_minus	number of coding sequences on the - strand of the genomic sequence
n_cds_total	total number of coding sequences in the genomic sequence
bp_cdsA	total number of Adenines in the coding sequences
bp_cdsT	total number of Thymines in the coding sequences
bp_cdsC	total number of Cytosines in the coding sequences
bp_cdsG	total number of Guanines in the coding sequences

bp_cds_total	total number of bases in the coding sequences
fr_cdsA	frequency of Adenine nucleobase in the coding sequences
fr_cdsT	frequency of Thymine nucleobase in the coding sequences
fr_cdsC	frequency of Cytosine nucleobase in the coding sequences
fr_cdsG	frequency of Guanine nucleobase in the coding sequences
cds_topological_entropy_score	Topological score calculated on coding sequences
cds_chargaff_score_pf	Chargaff's second parity rule score calculated on coding sequences using PF method
cds_chargaff_score_ct	Chargaff's second parity rule score calculated on coding sequences using CT method
cds_shannon_score	Shannon’s score calculated on coding sequences
n_ncRNA_plus	number of ncRNAs on the + strand of the genomic sequence
n_ncRNA_minus	number of ncRNAs on the - strand of the genomic sequence
n_ncRNA_total	total number of ncRNAs in the genomic sequence
bp_ncRNA_A	total number of Adenines in the ncRNA sequences
bp_ncRNA_T	total number of Thymines in the ncRNA sequences
bp_ncRNA_C	total number of Cytosines in the ncRNA sequences
bp_ncRNA_G	total number of Guanines in the ncRNA sequences
bp_ncRNA_total	total number of bases in the ncRNA sequences
fr_ncRNA_A	frequency of Adenine nucleobase in the ncRNA sequences
fr_ncRNA_T	frequency of Thymine nucleobase in the ncRNA sequences
fr_ncRNA_C	frequency of Cytosine nucleobase in the ncRNA sequences
fr_ncRNA_G	frequency of Guanine nucleobase in the ncRNA sequences
ncRNA_topological_entropy_score	Topological score calculated on ncRNA sequences

ncRNA_chargaff_score_pf	Chargaff's second parity rule score calculated on ncRNA sequences using PF method
ncRNA_chargaff_score_ct	Chargaff's second parity rule score calculated on ncRNA sequences using CT method
ncRNA_shannon_score	Shannon’s score calculated on ncRNA sequences
n_tRNA_plus	number of tRNAs on the + strand of the genomic sequence
n_tRNA_minus	number of tRNAs on the - strand of the genomic sequence
n_tRNA_total	total number of tRNAs in the genomic sequence
bp_tRNA_A	total number of Adenines in the tRNA sequences
bp_tRNA_T	total number of Thymines in the tRNA sequences
bp_tRNA_C	total number of Cytosines in the tRNA sequences
bp_tRNA_G	total number of Guanines in the tRNA sequences
bp_tRNA_total	total number of bases in the tRNA sequences
fr_tRNA_A	frequency of Adenine nucleobase in the tRNA sequences
fr_tRNA_T	frequency of Thymine nucleobase in the tRNA sequences
fr_tRNA_C	frequency of Cytosine nucleobase in the tRNA sequences
fr_tRNA_G	frequency of Guanine nucleobase in the tRNA sequences
tRNA_topological_entropy_score	Topological score calculated on tRNA sequences
tRNA_chargaff_score_pf	Chargaff's second parity rule score calculated on tRNA sequences using PF method
tRNA_chargaff_score_ct	Chargaff's second parity rule score calculated on tRNA sequences using CT method
tRNA_shannon_score	Shannon’s score calculated on tRNA sequences
n_rRNA_plus	number of rRNAs on the + strand of the genomic sequence
n_rRNA_minus	number of rRNAs on the - strand of the genomic sequence
n_rRNA_total	total number of rRNAs in the genomic sequence
bp_rRNA_A	total number of Adenines in the rRNA sequences
bp_rRNA_T	total number of Thymines in the rRNA sequences
bp_rRNA_C	total number of Cytosines in the rRNA sequences

bp_rRNA_G	total number of Guanines in the rRNA sequences
bp_rRNA_total	total number of bases in the rRNA sequences
fr_rRNA_A	frequency of Adenine nucleobase in the rRNA sequences
fr_rRNA_T	frequency of Thymine nucleobase in the rRNA sequences
fr_rRNA_C	frequency of Cytosine nucleobase in the rRNA sequences
fr_rRNA_G	frequency of Guanine nucleobase in the rRNA sequences
rRNA_topological_entropy_score	Topological score calculated on rRNA sequences
rRNA_chargaff_score_pf	Chargaff's second parity rule score calculated on rRNA sequences using PF method
rRNA_chargaff_score_ct	Chargaff's second parity rule score calculated on rRNA sequences using CT method
rRNA_shannon_score	Shannon's score calculated on rRNA sequences
