# Supplementary material for: Machine learning classification of archaea and bacteria identifies novel predictive genomic features
Source: BMC Genomics. 2024 Oct 14;25:955. doi: 10.1186/s12864-024-10832-y (PMC11472548; doi:10.1186/s12864-024-10832-y)
Supplement: Supplementary file 2 [file 12864_2024_10832_MOESM2_ESM.pdf]

S1 Appendix. Tuning details of the four machine learning algorithms.

Regularized logistic regression with Ridge, Lasso or Elastic-Net penalties (method = “glmnet”)

Resampling results across tuning parameters:

alpha	lambda	Accuracy	Kappa
0.10	0.0002690445	0.9962441	0.9569925
0.10	0.0026904450	0.9934183	0.9258833
0.10	0.0269044497	0.9877712	0.8664859
0.55	0.0002690445	0.9962441	0.9569925
0.55	0.0026904450	0.9948357	0.9424624
0.55	0.0269044497	0.9854193	0.8427986
**1.00**	**0.0002690445**	**0.9971831**	**0.9669511**
1.00	0.0026904450	0.9957702	0.9497503
1.00	0.0269044497	0.9807200	0.7921588

Accuracy was used to select the optimal model using the largest value.
The final values used for the model were alpha = 1 and lambda = 0.0002690445.

Random Forest (method = “rf”)

Resampling results across tuning parameters:

mtry	Accuracy	Kappa
2	0.9957746	0.9418165
**12**	**0.9971831**	**0.9632442**
23	0.9967114	0.9577348

Accuracy was used to select the optimal model using the largest value.
The final value used for the model was mtry = 12.

Support Vector Machines with Radial Basis Function Kernel (method = “svmRadial”)

Resampling results across tuning parameters:

C	Accuracy	Kappa
0.25	0.9985915	0.9828577
0.50	0.9990610	0.9883659
**1.00**	**0.9995305**	**0.9938740**

Tuning parameter 'sigma' was held constant at a value of 0.05540714
Accuracy was used to select the optimal model using the largest value.
The final values used for the model were sigma = 0.05540714 and C = 1.

Neural Networks (method = “nnet”)

Resampling results across tuning parameters:

size	decay	Accuracy	Kappa
**1**	**0e+00**	**0.9995283**	**0.9930900**
1	1e-04	0.9957636	0.9471167
1	1e-01	0.9971765	0.9654970
3	0e+00	0.9962331	0.9532427
3	1e-04	0.9971720	0.9630022
3	1e-01	0.9971765	0.9654970
5	0e+00	0.9971787	0.9662811
5	1e-04	0.9981176	0.9764753
5	1e-01	0.9971765	0.9654970

Accuracy was used to select the optimal model using the largest value.
The final values used for the model were size = 1 and decay = 0.
