# Supplementary material for: Machine learning classification of archaea and bacteria identifies novel predictive genomic features
Source: BMC Genomics. 2024 Oct 14;25:955. doi: 10.1186/s12864-024-10832-y (PMC11472548; doi:10.1186/s12864-024-10832-y)
Supplement: Supplementary file 3 [file 12864_2024_10832_MOESM3_ESM.pdf]

S1 Fig. Results from principal component analysis of 77 genomic features extracted by the GBRAP tool from 2655 genome sequences of bacteria and archaea. A) scree plot of principal components in decreasing order of percentage of total variance explained (the first 3 PCs account for 61.5% of the total variance in the data); B) correlation plot of the 77 genomic features; C) multidimensional scaling plot of Euclidean distances between microorganisms (bacteria, archaea) based on the matrix of genomic features: first two dimensions.

A)

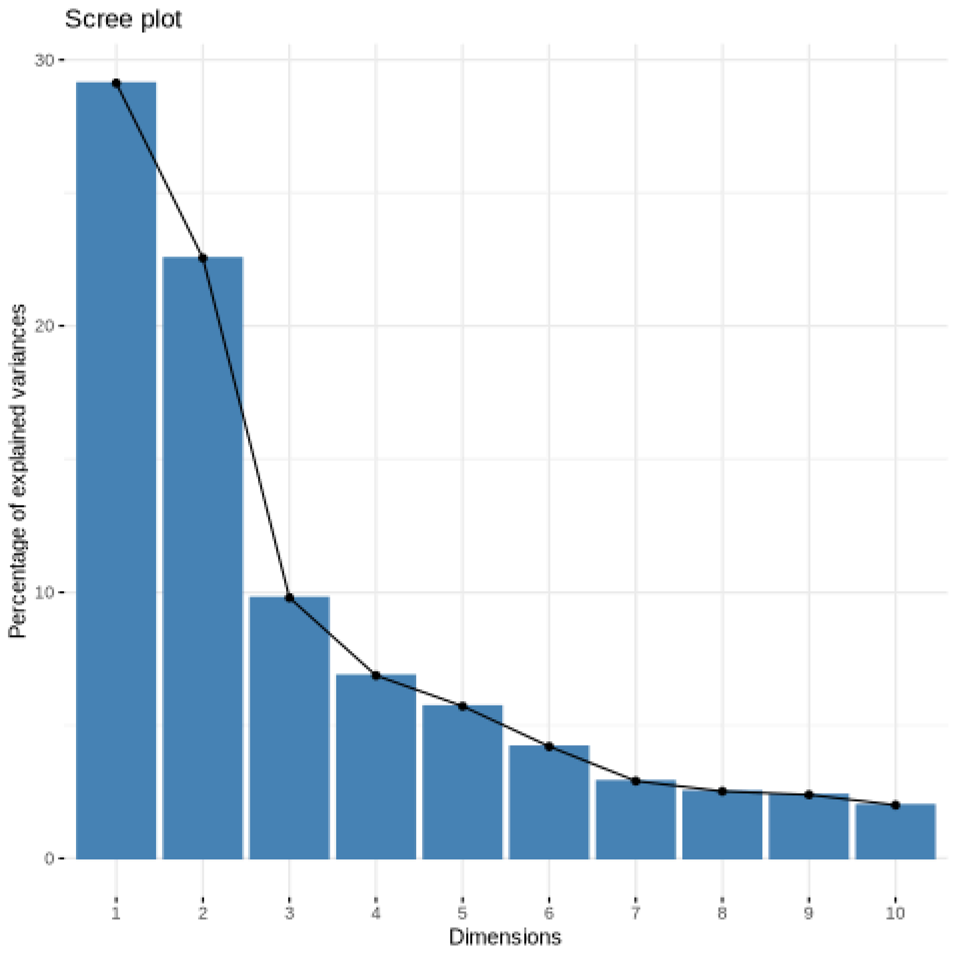

B)

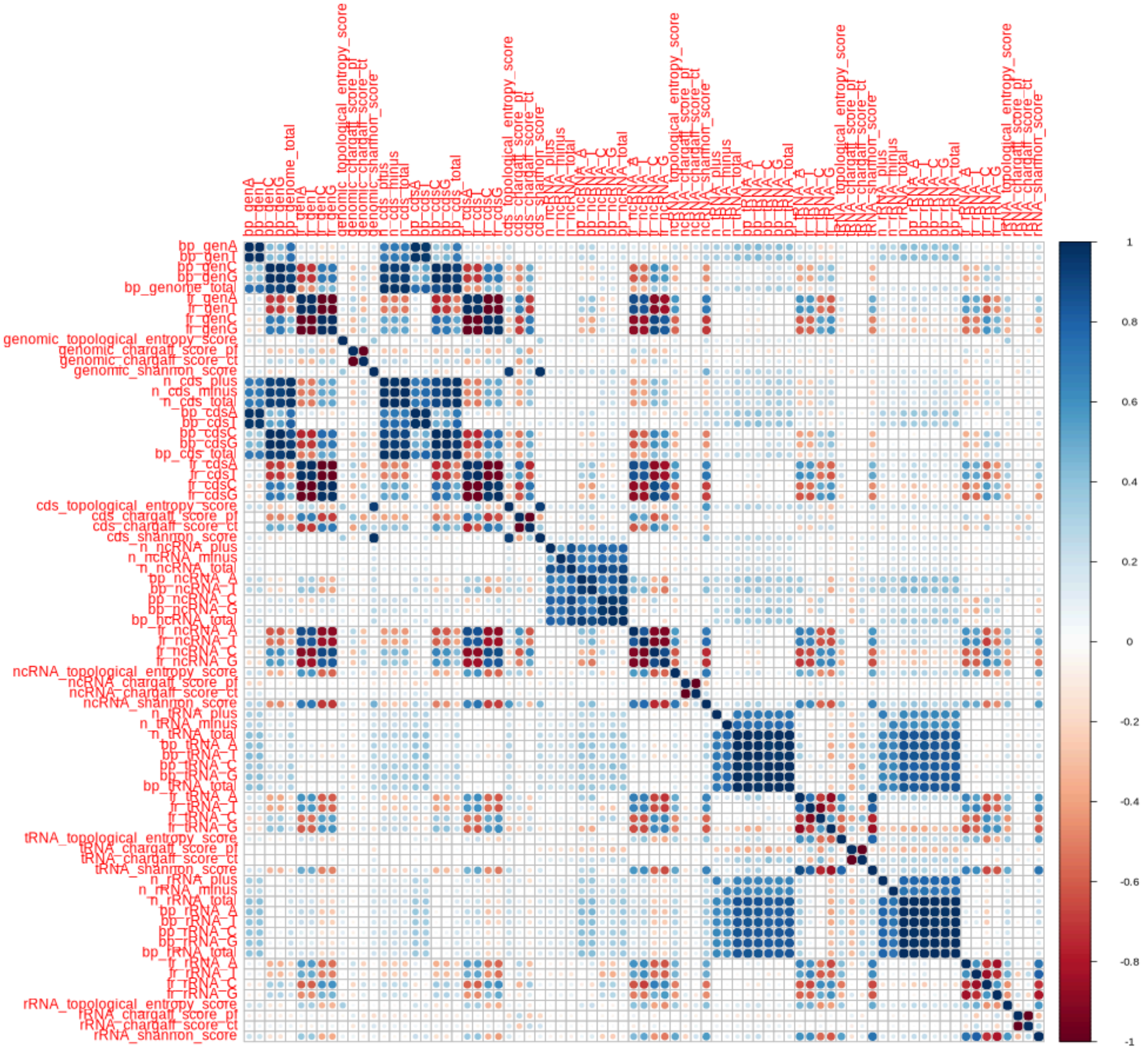
bp_genA
bp_genT
bp_genC
bp_genG
bp_genome_total
fr_genA
fr_genT
fr_genC
fr_genG
genomic_topological_entropy_score
genomic_chargaff_score_pf
genomic_chargaff_score_ct
genomic_shannon_score
n_cds_plus
n_cds_minus
n_cds_total
bp_cdsA
bp_cdsT
bp_cdsC
bp_cdsG
bp_cds_total
fr_cdsA
fr_cdsT
fr_cdsC
fr_cdsG
cds_topological_entropy_score
cds_chargaff_score_pf
cds_chargaff_score_ct
cds_shannon_score
n_ncRNA_plus
n_ncRNA_minus
n_ncRNA_total
bp_ncRNA_A
bp_ncRNA_T
bp_ncRNA_C
bp_ncRNA_G
bp_ncRNA_total
fr_ncRNA_A
fr_ncRNA_T
fr_ncRNA_C
fr_ncRNA_G
ncRNA_topological_entropy_score
ncRNA_chargaff_score_pf
ncRNA_chargaff_score_ct
ncRNA_shannon_score
n_tRNA_plus
n_tRNA_minus
n_tRNA_total
bp_tRNA_A
bp_tRNA_T
bp_tRNA_C
bp_tRNA_G
bp_tRNA_total
fr_tRNA_A
fr_tRNA_T
fr_tRNA_C
fr_tRNA_G
tRNA_topological_entropy_score
tRNA_chargaff_score_pf
tRNA_chargaff_score_ct
tRNA_shannon_score
n_rRNA_plus
n_rRNA_minus
n_rRNA_total
bp_rRNA_A
bp_rRNA_T
bp_rRNA_C
bp_rRNA_G
bp_rRNA_total
fr_rRNA_A
fr_rRNA_T
fr_rRNA_C
fr_rRNA_G
rRNA_topological_entropy_score
rRNA_chargaff_score_pf
rRNA_chargaff_score_ct
rRNA_shannon_score
1
0.8
0.6
0.4
0.2
0
-0.2
-0.4
-0.6
-0.8
-1

C)

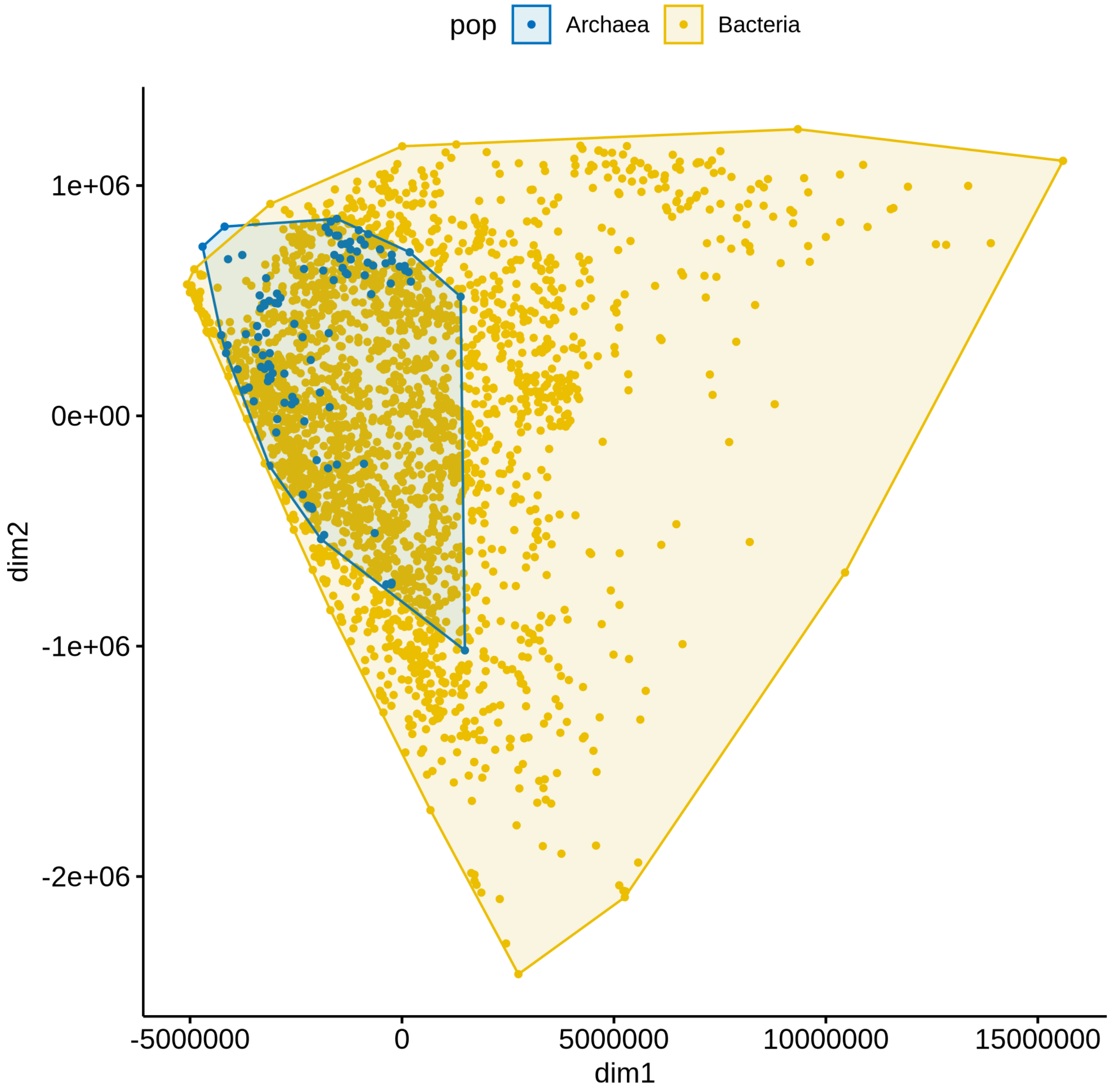
pop
Archaea
Bacteria
1e+06
0e+00
-1e+06
-2e+06
dim2
-5000000
0
5000000
10000000
15000000
dim1
