# Supplementary material for: Machine learning classification of archaea and bacteria identifies novel predictive genomic features
Source: BMC Genomics. 2024 Oct 14;25:955. doi: 10.1186/s12864-024-10832-y (PMC11472548; doi:10.1186/s12864-024-10832-y)
Supplement: Supplementary file 4 [file 12864_2024_10832_MOESM4_ESM.pdf]

S2 Table. Median values in Archaea and Bacteria for the 23 features selected by RFE (recursive feature elimination). The p-values come from Wilcoxon test.

feature	Archaea	Bacteria	p-value
bp_ncRNA_A	141	151	1.14E-03
bp_ncRNA_C	208	166	1.56E-08
bp_ncRNA_G	256	206	5.08E-10
bp_ncRNA_total	675	653	6.49E-02
bp_rRNA_A	2226	5958.5	1.04E-47
bp_rRNA_G	2835	6968	6.37E-43
bp_rRNA_T	1623	4721.5	4.49E-48
cds_chargaff_score_ct	0.802	0.8463	1.82E-18
cds_chargaff_score_pf	0.2302	0.17495	1.21E-19
fr_rRNA_C	0.28	0.24	1.10E-45
fr_rRNA_G	0.32	0.31	5.64E-05
fr_tRNA_A	0.18	0.2	2.10E-55
fr_tRNA_C	0.3	0.28	1.78E-45
fr_tRNA_G	0.33	0.31	2.39E-46
fr_tRNA_T	0.2	0.22	1.33E-42
n_ncRNA_total	2	3	5.07E-34
ncRNA_topological_entropy_score	0.8798	0.8893	2.19E-03
rRNA_chargaff_score_ct	0.8063	0.78995	2.02E-08
rRNA_chargaff_score_pf	0.2174	0.23925	9.08E-09
tRNA_chargaff_score_ct	0.8428	0.8618	7.10E-12
tRNA_chargaff_score_pf	0.1815	0.15395	8.52E-16
tRNA_shannon_score	1.9395	1.9654	1.28E-55
tRNA_topological_entropy_score	0.8747	0.8883	2.99E-67
