# Supplementary material for: Machine learning classification of archaea and bacteria identifies novel predictive genomic features
Source: BMC Genomics. 2024 Oct 14;25:955. doi: 10.1186/s12864-024-10832-y (PMC11472548; doi:10.1186/s12864-024-10832-y)

S2 Fig. This figure illustrates the topological entropy of CDS (coding sequences) for different taxonomic groups of microorganisms, showing that prokaryotic CDS (both archaea and bacteria) exhibit higher entropy compared to eukaryotic CDS. This indicates that prokaryotic CDS are more random than those of eukaryotes. Additionally, the entropy levels of archaeal CDS are intermediate, falling between those of bacterial and eukaryotic CDS.

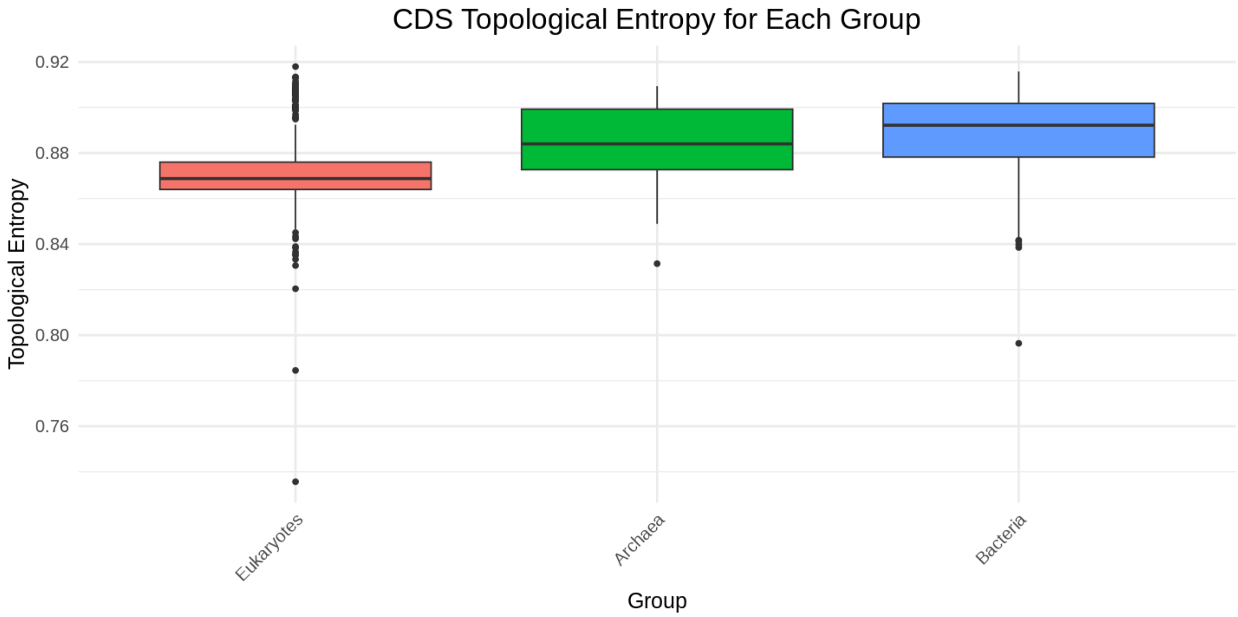

Supplement: Supplementary file 5 [file 12864_2024_10832_MOESM5_ESM.pdf]
