# Supplementary material for: Machine learning classification of archaea and bacteria identifies novel predictive genomic features
Source: BMC Genomics. 2024 Oct 14;25:955. doi: 10.1186/s12864-024-10832-y (PMC11472548; doi:10.1186/s12864-024-10832-y)

S3 Fig. Receiver operating characteristic (ROC) curves of four machine learning methods [Regularized logistic regression (RLR), Random Forest (RF), Support vector machines (SVM) and Neural networks (NN)] run for predicting the domain (Archaea or Bacteria) on the test set. In each plot, area under the curve (AUC) and 95% confidence interval are reported.

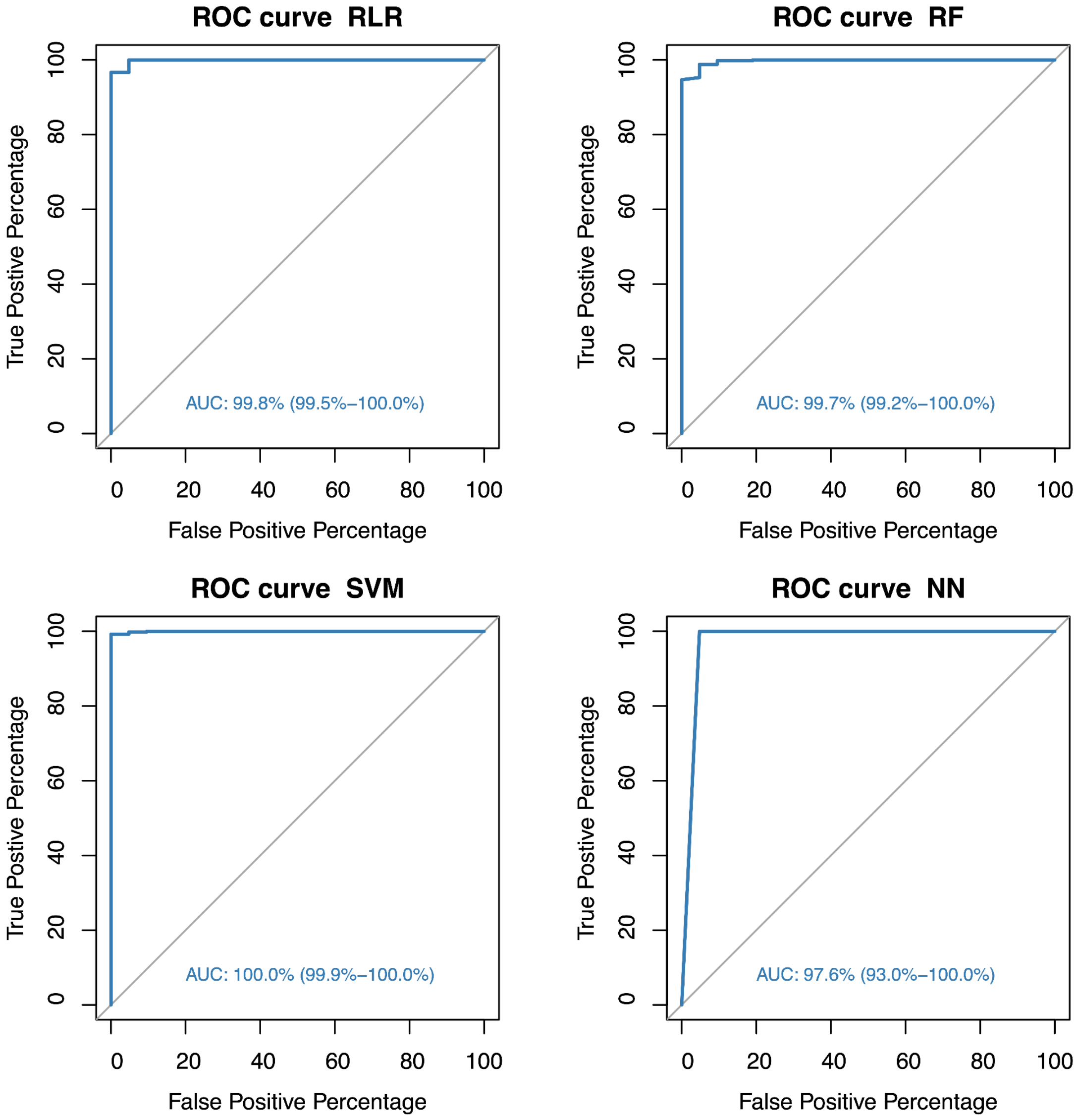

Supplement: Supplementary file 6 [file 12864_2024_10832_MOESM6_ESM.pdf]
